# Liver Transplantation in Recipients With a Positive Crossmatch: A Retrospective Single-Center Match-Pair Analysis

**DOI:** 10.3389/ti.2023.11062

**Published:** 2023-03-02

**Authors:** Felix J. Krendl, Margot Fodor, Franka Messner, Agnes Balog, Anja Vales, Benno Cardini, Thomas Resch, Manuel Maglione, Christian Margreiter, Marina Riedmann, Hanno Ulmer, Dietmar Öfner, Rupert Oberhuber, Stefan Schneeberger, Annemarie Weissenbacher

**Affiliations:** ^1^ Department of Visceral, Transplant and Thoracic Surgery, Center of Operative Medicine, Medical University of Innsbruck, Innsbruck, Austria; ^2^ Blood Transfusion Center, Innsbruck, Austria; ^3^ Department of Medical Statistics, Informatics, and Health Economics, Medical University of Innsbruck, Innsbruck, Austria

**Keywords:** liver transplantation, biliary complications, graft survival, crossmatch, CMV

## Abstract

A positive crossmatch (XM+) is considered a contraindication to solid abdominal organ transplantation except liver transplantation (LT). Conflicting reports exist regarding the effects of XM+ on post-transplant outcomes. The goal of this retrospective single-center analysis is to evaluate the influence of XM+ on relevant outcome parameters such as survival, graft rejection, biliary and arterial complications. Forty-nine adult patients undergoing LT with a XM+ between 2002 and 2017 were included. XM+ LT recipients were matched 1:2 with crossmatch negative (XM−) LT recipients based on the balance of risk (BAR) score. Patient and graft survival were compared using Kaplan-Meier survival analysis and the log-rank test. Comparative analysis of clinical outcomes in XM+ and XM− groups were conducted. Patient and graft survival were similar in XM+ and XM− patients. Rejection episodes did not differ either. Recipients with a strong XM+ were more likely to develop a PCR+ CMV infection. A XM+ was not associated with a higher incidence of biliary or arterial complications. Donor age, cold ischemia time, PCR+ CMV infection and a rejection episode were associated with the occurrence of ischemic type biliary lesions. A XM+ has no effects on patient and graft survival or other relevant outcome parameters following LT.

## Graphical Abstract

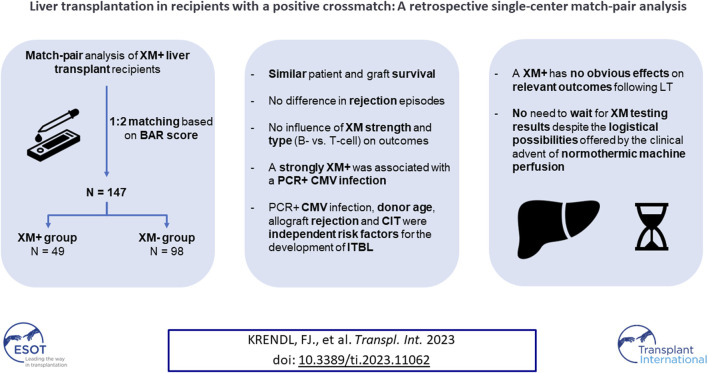

## Introduction

A positive crossmatch (XM+) is usually considered a contraindication to all solid abdominal organ transplantations except liver transplantation (LT) (1, 2). Therefore, crossmatch testing is mandatory before pancreas, intestinal and kidney transplantation (3). However, in the context of LT the effect of a XM+ on post-transplant outcomes remains ill-defined and LT is commonly performed regardless of the crossmatch testing results, often even before these results become available (3–7).

Compared to other abdominal organs, the liver seems to be in a privileged immunological situation due to its dual afferent blood supply, its unique antigenic sinusoidal vasculature line by Kupffer cells and its ability to absorb preformed donor specific antibodies (DSAs) by secreting soluble antigens (8–10). Reports of combined liver and kidney transplantations in the presence of a XM+ in which the recipient became XM− within hours following transplantation underline the liver’s impressive immunologic capabilities (9, 11, 12).

Still, some authors suggest a link between inferior patient and graft survival and a higher rate of postoperative complications following LT in the presence of a XM+ (8, 13–18). Others, however, were not able to duplicate those findings (6, 10, 12, 19–25). Yet, focusing on a XM+ alone might not tell the full story as XM strength (26) and type (T cell vs. B cell) may play a significant role concerning post-transplant outcomes (3, 5, 17, 18). Fittingly, a T cell but not B cell dependent XM+ was reported to be associated with impaired graft survival (3). Historically, LT was essentially an emergency surgical procedure in order to keep cold ischemia time (CIT) short. While it seemed unthinkable to postpone a LT until crossmatch testing results become available only a few years ago, the advent of machine perfusion has changed clinical practice (27). Machine preservation offers the possibility to optimize transplant conditions including immunologic risk stratification pre-transplant. Considering these implications, it seems worthwhile to explore whether a XM+ influences post-transplant outcomes. Previous studies on this subject were hampered by a small number of patients and mostly lacked adequate controls and comparisons (15, 16, 26, 28, 29).

The aim of this match-pair analysis is to evaluate the influence of a XM+, including XM strength and type, on relevant clinical outcome parameters such as patient and graft survival, rejection episodes, biliary and arterial complications.

## Patients and Methods

### Study Population and Study Design

At the Medical University of Innsbruck, crossmatch testing is routinely performed for LT recipients. All adult patients who underwent XM+ deceased donor LT from donation after brain dead (DBD) donors between 2002 and 2017 were included. A 1:2 match-pair analysis was conducted, with patients who underwent LT with a negative crossmatch (XM−) serving as controls. Matching was performed based on the balance of risk (BAR) score (30, 31).

The study was conducted in accordance with the guidelines of the Declaration of Helsinki and approved by the Institutional Review Board; protocol code 1034/2022. The results were reported according to the STROBE guidelines (32).

### Immunosuppression and Postoperative Care

The standard immunosuppressive (IS) regimen for LT recipients at our center consisted of the following: Induction therapy with an intra-operative bolus of 500 mg methylprednisolone. As part of the PROTECT (33) and DIAMOND (34) trials, some patients received induction therapy with an interleukin 2 (IL2) antibody. Postoperatively patients received tacrolimus (Tac) (initial trough levels 6–8 ng/mL, gradually decreased to 6 ng/mL at 6 months, and 4–6 ng/mL at 12 months) and either mycophenolate mofetil (MMF) (1,000 mg twice daily) or mycophenolic acid (MPA) (720 mg twice daily). Steroids were gradually tapered to 5 mg prednisolone per day as part of the maintenance therapy. Complete steroid withdrawal was considered on an individual basis considering the side effect profile as well as the patient’s immunologic risk. Reasons to divert from our standard protocol were related to recipient factors. Conversion from Tac to cyclosporine A (CsA) was considered in case of long-QT syndrome, or tacrolimus associated neurotoxicity. MMF/MPA was switched to azathioprine (Aza) in case of gastrointestinal side effects or to avoid the teratogenic potential in female patients wishing to conceive.

### Definitions

#### Crossmatch

All recipient sera were tested for cytotoxic antibodies against donor lymphocytes (CDC crossmatch). For the XM to be deemed positive more than 15% cytolysis had to be present. Additionally, a XM was defined as weakly positive when cytolysis ranged between 15% and 50% and strongly positive when cytolysis exceeded 50%. Cytotoxic cross-matching activity was tested before and after treatment with dithiothreitol (DTT) which inactivates IgM antibodies (35, 36). For XM strength analysis the post DTT treatment value was employed. In addition to XM strength, the XM type (T cell dependent vs. B cell dependent) was recorded.

#### Graft Loss and Graft Dysfunction

Graft loss was defined as patient death or the need for liver re-transplantation. Primary non-function was defined as peak AST ≥3000 IU/L plus at least one of the following criteria: INR ≥2.5, serum lactate ≥4 mmol/L and total bilirubin ≥10 mg/dL (values measured on postoperative day 3, biliary obstruction being excluded). Early allograft dysfunction (EAD) was defined according to the Olthoff criteria (37).

#### Rejections

Acute rejection was defined as biopsy proven rejection which required steroid bolus treatment (38). Steroid bolus treatment consisted of an intravenous steroid pulse of 500 mg methylprednisolone for three consecutive days. Chronic rejection was defined based on persistent laboratory abnormalities and histological confirmation (38).

#### Biliary Complications

Biliary complications were classified as bile duct leaks, biliary cast syndrome, anastomotic stenosis (AS) and non-anastomotic stenosis (NAS). Ischemic type biliary lesions (ITBL) were defined as NAS with or without biliary cast formation in the absence of hepatic artery stenosis or thrombosis (39–41).

#### Extended Criteria Donors

ECDs were defined according to the Eurotransplant Manual, Chapter 9: The Donor (42).

### Outcomes

The primary outcome was patient and graft survival. Secondary outcomes included incidence and risk factors for rejection episodes as well as incidence, risk factors and type of biliary and arterial complications.

### Statistical Analysis

A 1:2 optimal pair matching was performed with the goal of minimizing the absolute pairwise distances in the matched sample (median BAR score values XM+ 8.0 vs. XM− 7.5). For descriptive analysis, categorical variables were summarized with the help of absolute and relative (percentages) frequencies, continuous variables were summarized with means and standard deviation (SD) or medians and interquartile range (IQR) as appropriate. Comparative analysis of clinical outcomes in the XM+ and XM− group was conducted using the Chi-square or Fisher’s exact test (if one or more cells had an expected count of less than five) for categorical variables. The Mann–Whitney U test was used to compare continuous, not normally distributed variables. Any variable having a significant univariate test (*p*-value cut-off point of 0.25 based on the Wald test) was selected as a candidate for the multivariate analysis (43). Uni- and multivariate analyses were performed for the primary and secondary endpoints starting with a univariate analysis of each variable. Kaplan-Meier survival analysis was performed to compare patient and graft survival between XM+ patients and XM− patients using the log-rank test. Multivariate analysis for patient and graft survival endpoints was performed with Cox proportional hazards regression analysis. Logistic regression analysis was used to assess the effects of clinical parameters on secondary endpoints. Statistical analysis was conducted with SPSS (IBM SPSS Statistics for Windows, Version 26.0. Armonk, NY: IBM Corp).

## Results

### Recipient Characteristics

Forty-nine patients undergoing LT with a XM+ were matched 1:2 with XM− patients. Matching was performed based on the BAR score. The indications for LT and recipient demographics are presented in Table 1. The median recipient age was 58.0 years in the XM+ group compared to 59.0 years in the XM− group (*p* = 0.526). Patients in the XM+ group were more likely to be female [XM+ 36.7% (18 of 49) vs. XM− 19.4% (19 of 98), *p* = 0.022], have a lower BMI [XM+ 23.8 (21.6–27.1) vs. XM− 26.5 (23.9–29.1), *p* = 0.003] and a higher MELD score [XM+ 16.0 (12.0–21.0) vs. XM− 13.5 (8.8–17.0), *p* = 0.014] compared to patients in the XM− group. The groups were similar in terms of AB0 blood groups (*p* = 0.769), CMV mismatching (*p* = 0.228) and median follow-up (*p* = 0.304). Patients in the XM+ group had more commonly received a previous LT [XM+ 14.3% (7 of 49) vs. XM− 4.1% (4 of 98), *p* = 0.042]. The overall use of induction therapy was similar between the groups [XM+ 61.2% (30 of 49) vs. XM− 62.2% (61 of 98), *p* = 0.904]. However, XM+ patients more often received antibody induction with ATG [XM+ 4.1% (2 of 49) vs. XM− 0% (0 of 98), *p* = 0.110], although not statistically significant, and alemtuzumab [XM+ 8.2% (4 of 49) vs. XM− 1.0% (1 of 98), *p* = 0.042]. Yet, in a subgroup analysis, antibody induction had no significant influence on any of the explored outcome parameters including patient and graft survival.

**TABLE 1:** Recipient characteristics in the matched cohort.

	All N = 147	XM+ n = 49	XM− n = 98	*p*-value
Age (years)	59.0 (54.0–65.0)	58.0 (53.5–64.0)	59.0 (54.0–65.0)	0.526
Sex				*0.022*
Female	37 (25.2)	18 (36.7)	19 (19.4)	
Male	110 (74.8)	31 (63.3)	79 (80.6)	
BMI (kg/m^2^)	25.9 (22.9–28.8)	23.8 (21.6–27.1)	26.5 (23.9–29.1)	*0.003*
MELD score	16.0 (9.0–18.0)	16.0 (12.0–21.0)	13.5 (8.8–17.0)	*0.014*
Indication for LT				
AFLD	58 (39.5)	16 (32.7)	42 (42.9)	0.233
NAFLD	21 (14.3)	8 (16.3)	13 (13.3)	0.617
PBC	7 (4.8)	3 (6.1)	4 (4.1)	0.686
PSC	4 (2.7)	0 (0.0)	4 (4.1)	0.302
AIH	4 (2.7)	3 (6.1)	1 (1.0)	0.108
Tumor	62 (42.2)	13 (26.5)	49 (50.0)	*0.007*
Re - Tx	11 (7.5)	7 (14.3)	4 (4.1)	*0.042*
Induction (yes/no)	91 (61.9)	30 (61.2)	61 (62.2)	0.904
IL2	83 (57.2)	24 (49.0)	59 (61.5)	0.151
ATG	2 (1.4)	2 (4.1)	0 (0.0)	0.110
Alemtuzumab	5 (3.4)	4 (8.2)	1 (1.0)	*0.042*
Missing	1 (0.7)	0 (0.0)	1 (1.0)	
ABO blood group				0.769
A	58 (39.5)	22 (44.9)	36 (36.7)	
B	14 (9.5)	4 (8.2)	10 (10.2)	
0	61 (41.5)	18 (36.7)	43 (43.9)	
AB	14 (9.5)	5 (10.2)	9 (9.2)	
CMV mismatch				0.228
D+/R-	35 (23.8)	9 (19.6)	26 (26.8)	
D-/R+	38 (25.9)	10 (21.7)	28 (28.9)	
D+/R+	54 (36.7)	23 (50.0)	31 (32.0)	
D-/R-	16 (10.9)	4 (8.7)	12 (12.4)	
Missing	4 (2.7)	3 (6.1)	1 (1.0)	
Median follow-up (months)	60.2 (25.0–98.6)	70.7 (33.1–108.0)	57.9 (23.8–96.8)	0.304

Values are presented as medians or absolute numbers with IQRs and percentages in parentheses. Italic values show significant *p*-values. AFLD, alcoholic fatty liver disease; AIH, autoimmune hepatitis; ATG, anti-thymocyte globulin; BAR, balance of risk; BMI, body mass index; CMV, cytomegalovirus; COD, cause of death; CVA, cerebrovascular accident; ET-DRI, Eurotransplant donor risk index; MELD, model for end-stage liver disease; NAFLD, non-alcoholic fatty liver disease. IL2, interleukin 2. IQR, interquartile range; PBC, primary biliary cirrhosis; PSC, primary sclerosing cholangitis; PCR, polymerase chain reaction. Re-Tx, re-transplantation. SAB, subarachnoid hemorrhage; XM, crossmatch.

### Donor Characteristics

Donor age [XM+ 55.0 (41.5–65.5) vs. XM− 52.0 (43.0–62.0), *p* = 0.639], and ET-DRI [XM+ 1.67 (1.40–1.91) vs. XM− 1.57 (1.39–1.86), *p* = 0.659] were similar between groups. The overall ET-DRI was 1.64, suggesting that very good quality grafts were used in this cohort. XM+ recipients more commonly received a graft from a female donor [XM+ female 61.2% (30 of 49) vs. XM− 42.9% (42 of 98), *p* = 0.036] and donor BMI was significantly lower in the XM+ group compared to the XM− group [XM+ 24.2 (22.6–26.2) vs. XM− 26.8 (23.9–29.8), *p* = 0.001]. Donor BMI and liver steatosis correlated directly with each other (*p* = 0.001). Anhepatic time [XM+ 51.0 (43.3–57.8) vs. XM− 57.0 (47.8–65.3), *p* = 0.007] and warm ischemic time (WIT) [XM+ 41.5 (36.0–51.0) vs. XM− 47.5 (41.0–56.0), *p* = 0.008] were significantly shorter in the XM+ group. University of Wisconsin (UW) solution was more commonly used as a preservation solution in the XM+ compared to the XM− group [XM+ 37.5% (18 of 49) vs. XM− 19.4% (19 of 98), *p* = 0.018] (Table 2).

**TABLE 2:** Donor characteristics and operative data in the matched cohort.

	All N = 147	XM+ *n* = 49	XM− n = 98	*p*-value
Age (years)	53.0 (42.0–62.3)	55.0 (41.5–65.5)	52.0 (43.0–62.0)	0.639
Sex				*0.036*
Female	72 (49.0)	30 (61.2)	42 (42.9)	
Male	75 (51.0)	19 (38.8)	56 (57.1)	
BMI (kg/m^2^)	25.7 (22.9–29.0)	24.2 (22.6–26.2)	26.8 (23.9–29.8)	*0.001*
COD				0.132
Trauma	37 (25.3)	16 (32.6)	21 (21.4)	
Anoxia	11 (7.5)	1 (2.0)	10 (10.2)	
CVA	96 (65.3)	31 (63.2)	65 (66.3)	
Other	2 (1.3)	0 (0.0)	2 (2.0)	
Missing	1 (0.7)	1 (2.0)	0 (0.0)	
ECD	109 (74.7)	33 (68.8)	76 (77.6)	0.251
Preservation				*0.018*
UW	37 (25.3)	18 (37.5)	19 (19.4)	
HTK	109 (74.7)	30 (62.5)	79 (80.6)	
Missing	1 (0.7)	1 (2.0)	0 (0.0)	
Anhepatic time (min)	54.0 (46.0–63.0)	51.0 (43.3–57.8)	57.0 (47.8–65.3)	*0.007*
WIT (min)	46.0 (39.0–55.0)	41.5 (36.0–51.0)	47.5 (41.0–56.0)	*0.008*
CIT (h)	8.6 (7.5–10.0)	8.8 (7.5–10.7)	8.4 (7.5–9.8)	0.316
ET-DRI	1.64 (1.40–1.88)	1.67 (1.40–1.91)	1.57 (1.39–1.86)	0.659

Values are presented as medians or absolute numbers with IQRs and percentages in parentheses; Italic values show significant *p*-values. BMI, body mass index; COD, cause of death; CVA, cerebrovascular accident; ECD, extended criteria donor; ET-DRI, Eurotransplant donor risk index; HTK, histidine-tryptophan-ketoglutarate. IQR, interquartile range; SAB, subarachnoid hemorrhage; UW, University of Wisconsin; WIT, warm ischemia time; XM, crossmatch.

### Early Allograft Dysfunction

The EAD rate was similar in XM+ and XM− patients [XM+ 24.5% (12 of 49) vs. XM− 37.8% (37 of 98), *p* = 0.138] (Table 3). XM strength or type had no influence on EAD rates. EAD, however, was associated with a positive CMV PCR. Univariate analysis showed recipient BMI, graft steatosis, donor gGT and XM type to be risk factors for the development of EAD. Considering these factors for multivariate analysis, only donor gGT remained as a statistically significant factor for the development of EAD (*p* = 0.045).

**TABLE 3:** Clinical outcomes and complications.

	All N = 147	XM+ n = 49	XM− n = 98	*p*-value
EAD	49 (33.3)	12 (24.5)	37 (37.8)	0.138
Rejection	17 (11.6)	7 (14.3)	10 (10.2)	0.466
Acute	12 (8.2)	5 (10.2)	7 (7.1)	0.535
Chronic	5 (3.4)	2 (4.1)	3 (3.1)	1.000
Biliary complications	61 (41.5)	21 (42.9)	40 (40.8)	0.813
Bile duct leaks	22 (15.0)	5 (10.2)	17 (17.3)	0.252
AS	37 (25.2)	14 (28.6)	23 (23.5)	0.502
NAS	15 (10.2)	6 (12.2)	9 (9.2)	0.563
ITBL	15 (10.2)	6 (12.2)	9 (9.2)	0.563
Casts	20 (13.6)	6 (12.2)	14 (14.3)	0.734
Arterial complications	13 (8.8)	2 (4.1)	11 (11.2)	0.220
Stenosis	2 (1.4)	0 (0.0)	2 (2.0)	0.553
Thrombosis	6 (4.1)	1 (2.0)	5 (5.1)	0.664
Dissection	6 (4.1)	1 (2.0)	5 (5.1)	0.664
CMV PCR +	31 (20.7)	14 (28.6)	17 (17.3)	0.116

Values are presented as absolute numbers with percentages in parentheses. AS, anastomotic stricture. XM+, positive crossmatch. XM−, negative crossmatch. CMV, cytomegalovirus; EAD, early allograft dysfunction; ITBL, ischemic type biliary lesion; NAS, non-anastomotic stricture; PCR, polymerase chain reaction; PNF, primary non-function.

### Rejection Episodes

Rejection episodes did not differ significantly between XM+ and XM− recipients [XM+ 14.3% (7 of 49) vs. XM− 10.2% (10 of 98), *p* = 0.466]. XM strength (*p* = 0.400) and type (*p* = 0.282) had no influence on the incidence of rejection episodes. Acute and chronic rejection rates were similar between groups [acute: XM+ 10.2% (5 of 49) vs. XM− 7.1% (7 of 98), *p* = 0.535; chronic: XM+ 4.1% (2 of 49) vs. XM− 3.1% (3 of 98), *p* = 1.000] (Table 3). Patients with a documented episode of allograft rejection tended to have more biliary complications than those without a rejection episode but that difference proved not to be statistically significant [58.8% (10 of 17) vs. 39.2% (51 of 130), *p* = 0.123]. Neither a CMV mismatch at LT (*p* = 0.546) nor a positive CMV PCR (*p* = 0.758) following LT was associated with the occurrence of rejection episodes.

### Biliary Complications

Of 147 patients, 61 (41.5%) developed biliary complications (Table 3). There was no significant difference in overall biliary complications between the XM+ and XM− group [XM+ 42.9% (21 of 49) vs. XM− 40.8% (40 of 98), *p* = 0.813]. Bile duct leaks occurred in 10.2% (XM+ 5 of 49) vs. 17.3% (XM− 17 of 98), (*p* = 0.252), anastomotic strictures in 28.6% (XM+ 14 of 49) vs. 23.5% (XM− 23 of 98), (*p* = 0.502), non-anastomotic strictures in 12.2% (XM+ 6 of 49) vs. 9.2% (XM− 9 of 98), (*p* = 0.563) and biliary casts in 12.2% (XM+ 6 of 49) vs. 14.3% (XM− 14 of 98), (*p* = 0.734). In all NAS cases the hepatic artery was patent without stenosis or thrombosis and therefore, according to the pre-specified definition, these cases were all recorded as ITBL. Recipients with ITBL received organs from older donors [donor age median 64.0 years (48.0–76.0) vs. 52.0 years (42.0–61.0), *p* = 0.027] and the duration of the CIT was longer [CIT median 9.8 h (8.3–11.4) vs. 8.5 h (7.5–9.8), *p* = 0.038]. ET-DRI, a score incorporating donor age and CIT, was also significantly higher for recipients with ITBL [ET-DRI median 2.00 (1.74–2.30) vs. 1.57 (1.38–1.84), *p* = 0.002]. An episode of active CMV replication was associated with the occurrence of ITBL (*p* = 0.018). Univariate analysis revealed donor age, CIT, ET-DRI, allograft rejection and active CMV replication as risk factors for the development of ITBL. Considering these parameters for multivariate analysis (except for ET-DRI, as this a composite parameter) the most independent significant factor was allograft rejection [OR 7.773 (95% CI 1.878–31.169), *p* = 0.005] followed by donor age [OR 1.076 (95% CI 1.021–1.135), *p* = 0.006], active CMV replication [OR 4.096 (95% CI 1.180–14.219), *p* = 0.026] and duration of CIT [OR 1.315 (95% CI 1.032–1.676), *p* = 0.027] (Table 4). Out of 61 patients with a biliary complication, 50 patients (82.0%) required an endoscopic retrograde cholangiopancreatography, 15 patients (24.6%) underwent a re-operation while 13 patients (21.3%) required a re-transplantation. Patients with an ITBL were more likely to require a re-transplantation [33.3% (5 of 15) vs. 12.1% (16 of 132), *p* = 0.042]. Overall, patients with biliary complications had a significantly higher graft loss rate compared to patients without biliary complications [47.5% (29 of 61) vs. 27.9% (24 of 86), *p* = 0.015]. Neither XM strength nor XM type were associated with the development of biliary complications or ITBL.

**TABLE 4:** Factors influencing ITBL - Multivariate analysis.

	OR	95% CI	*p*-value
Rejection	7.773	1.878–32.169	0.005
Donor Age	1.076	1.021–1.135	0.006
CMV PCR +	4.096	1.180–14.219	0.026
CIT	1.315	1.032–1.676	0.027

CI, confidence interval; CIT, cold ischemia time; CMV, cytomegalovirus; ITBL, ischemic type biliary lesions; OR, odds ratio; PCR, polymerase chain reaction.

### Arterial Complications

In total, 13 patients (8.8%) developed arterial complications. The incidence of arterial complications did not differ between patients with and those without a positive crossmatch [XM+ 4.1% (2 of 49) vs. XM− 11.2% (11 of 98), *p* = 0.220]. No difference regarding the incidence of hepatic artery thrombosis (HAT) was noted between groups [XM+ 2.0% (1 of 49) vs. XM− 5.1% (5 of 98), *p* = 0.664].

### CMV Infection

Overall, 20.7% of recipients developed a CMV infection (CMV PCR+). XM status was not associated with CMV PCR+ [XM+ 28.6% (14 of 49) vs. XM− 17.3% (17 of 98), *p* = 0.116]. Neither was XM type (*p* = 0.312). However, XM strength was associated with a CMV PCR+ [XM strong 50% (9 of 18) vs. XM weak 16.7% (5 of 30), *p* = 0.022]. CMV mismatch status at LT was associated with a subsequent CMV infection (D-/R- 0, D+/R- 4, D-/R+ 9, D+/R+ 17, *p* = 0.019).

### Patient and Graft Survival

**FIGURE 1:**
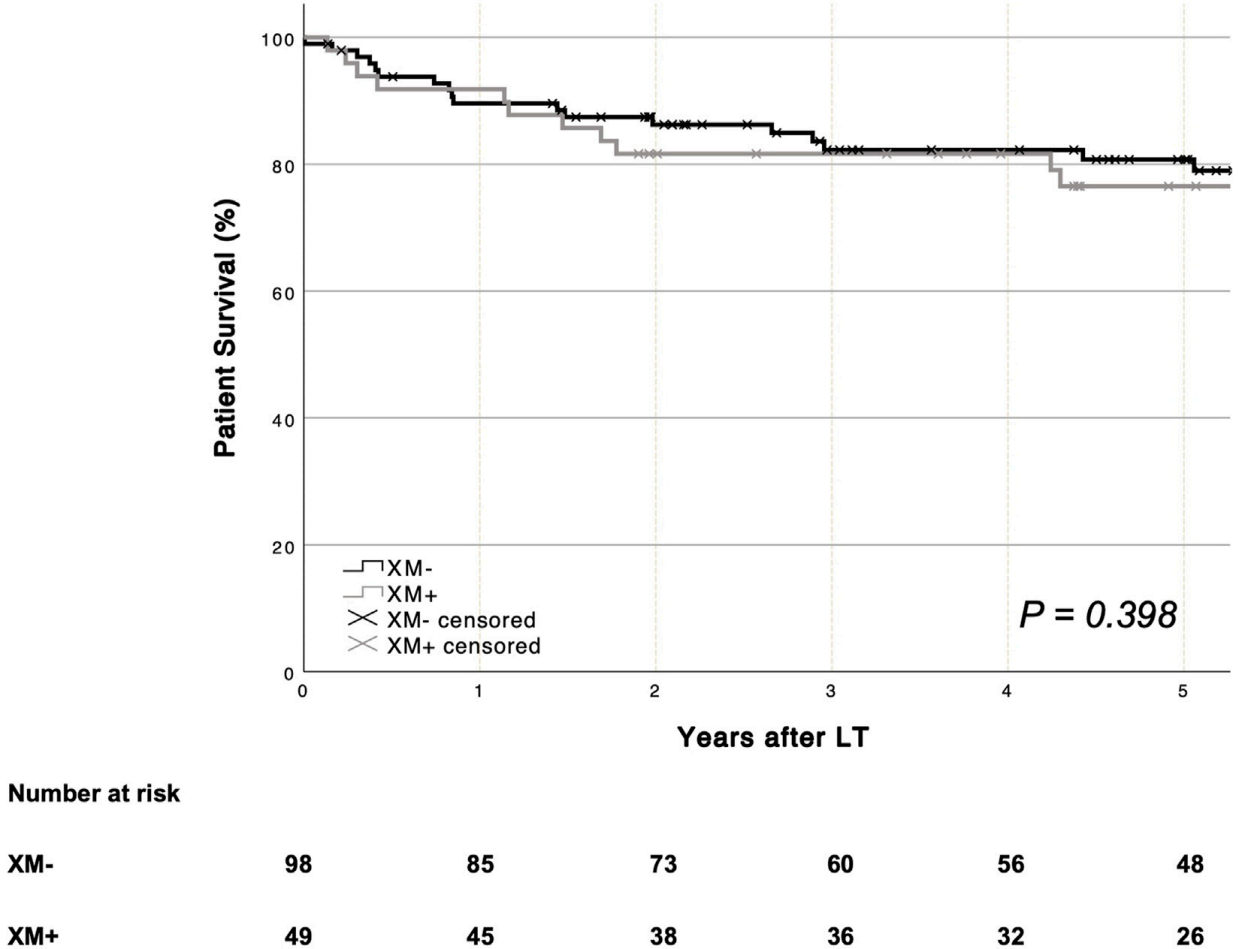
Overall patient survival was similar for recipients with and without a positive XM (log-rank *p* = 0.398). *LT, liver transplantation. XM, crossmatch.*

**FIGURE 2:**
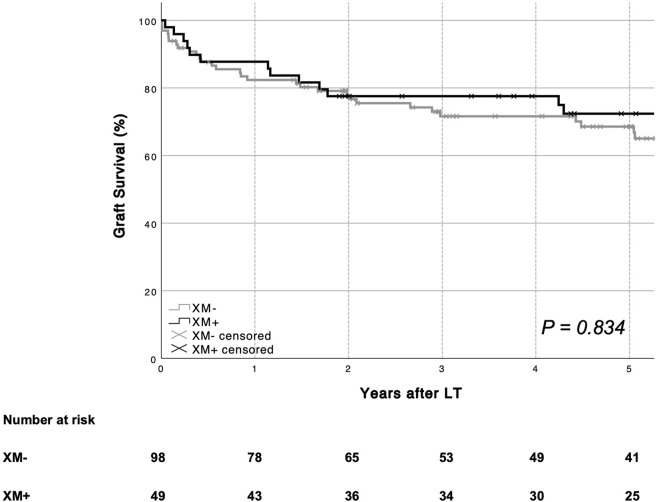
Overall graft survival according to XM status is shown. No difference in graft survival was seen based on recipient XM status (log-rank *p* = 0.834). *LT, liver transplantation. XM, crossmatch.*

Mean patient survival was similar in patients with (XM+) and those without (XM−) a positive crossmatch [XM+ 134.7 months (95% CI 107.5–161.9) vs. XM− 117.2 months (95% CI 105.5–128.9), *p* = 0.398]. One- and five-year patient survival rates are shown in Figure 1. Mean graft survival was comparable between groups [XM+ 114.4 months (95% CI 90.4–138.5) vs. XM− 97.8 months (95% CI 84.5–111.2), *p* = 0.834]. One- and five-year graft survival rates are shown in Figure 2. No single parameter, including XM strength or type, was found to affect patient or graft survival in univariate Cox regression analysis. Re-transplantation rates [XM+ 8.2% (4 of 49) vs. XM− 17.3 (17 of 98), *p* = 0.234] did not differ significantly between groups. One primary non-function (PNF) was recorded in the XM+ group, whereas no PNF occurred in the XM− group.

### Cause of Death

Overall, 37 out of 147 patients (25.2%) died during the observation period. Of those 37 patients, 13 (35%) died due to post-transplant malignancies, eight (22%) due to septic complications, six (16%) had recurrence of disease, six (16%) died of unknown causes, two (5%) died due to graft vs. host disease, one (3%) due to cardiovascular events and one (3%) due to other, non-specified reasons. Overall, 28 patients (76%) died with a functioning graft [XM+ 63% (10 of 16) vs. XM− 86% (18 of 21), *p* = 0.136].

## Discussion

This analysis comparing XM+ and XM− LT recipients over the course of a 16-year period demonstrated that a XM+ has no obvious effects on patient and graft survival and does not appear to influence any of the relevant clinical outcome parameters following LT such as rejection episodes, biliary or arterial complications. Furthermore, neither XM type nor strength had any influence on post-transplant outcomes.

Known risk factors for XM+ are female recipient sex, previous LT as well as immunologic indications for LT such as autoimmune hepatitis (AIH) (6, 14, 24). In contrast to an analysis by Ruiz et al. (6), patients with AIH were not at risk for a XM+ in our study. Considering that only four patients in our cohort underwent LT for AIH this finding needs to be viewed cautiously. However, similar to results reported by Ruiz et al. and others (8, 13, 24, 44, 45), we found a higher number of female recipients and re-transplantations in the XM+ group; attributable to previous pregnancies, blood transfusions during or in the aftermath of the primary transplant operation and sensitization caused by the initial graft itself. We also found the recipient BMI to be lower in XM+ recipients, which is in accordance with the finding that the XM+ group encompassed more female recipients.

A high rate of antibody induction (61.9%) was observed in the study cohort. This can be explained by the fact that our center took part in two IL2 antibody induction studies (PROTECT (33) and DIAMOND (34)) during the study period. While the overall antibody induction rate did not differ between XM+ and XM− negative patients, XM+ patients were more likely to receive alemtuzumab (although the absolute number was small). Interestingly, XM strength did not correlate with the use of antibody induction. However, XM strength did correlate with subsequent PCR+ CMV infections.

Overall, the number of rejection episodes was similar between our XM+ and XM− recipients. Previous studies have reported higher rejection rates in XM+ recipients (13, 14, 17, 46, 47). However, almost all of these studies used different definitions of what constitutes a positive XM. Charco et al. (13) and Bathgate et al. (14) defined a XM+ as cytolysis greater than 20%, while Takaya et al. (44) defined a XM+ as cytolysis of 50% or more. Furthermore, IS regimens differed between study centers (14, 17, 46, 47), and most of these studies were conducted decades ago when IS regimens were less intensive with lower CsA and Tac target levels. While originally reporting a higher complication rate in recipients with a XM+ Takaya et al. showed, in a follow-up study, that comparable outcomes can be achieved with a more intense IS regimen (48). The more intense IS regimen used in the follow-up study constitutes the standard IS regimen today at most transplant centers including ours (45). This might explain why, in more recent studies with more intense IS regimens, a XM+ had no influence on the occurrence of rejection episodes, patient and graft survival as well as overall complications (24, 25, 45, 49), which is in accordance with our observations. To the contrary: in a recent study by Ünlü et al. (50) LT recipients perceived to be at an increased immunologic risk received more intense IS leading to higher infectious complications without providing any graft or patient survival benefit. Considering the liver’s privileged immunologic status, a more intense IS for XM+ recipients might be unnecessary and even harmful. Accordingly, when analyzing their 20-year experience with XM+ LT recipients Ruiz et al. (6) found no association between a XM+ and graft complications as well as patient and graft survival.

Compared to previous studies (44, 51, 52), we were unable to find any association between a XM+, including XM strength and type, and the occurrence of biliary complications. Unsurprisingly, patients with biliary complications had a higher graft loss rate and patients with ITBL required re-transplantation more often. ITBL remain one of the most worrisome complications following LT. Immunologic factors have been implicated in the pathogenesis of ITBL in addition to ischemia reperfusion injury and bile salt toxicity (39). While a XM+ had no influence on ITBL development in our study, allograft rejection as well as a positive CMV PCR were associated with an increased risk for the development of ITBL in uni- and multivariate analysis; as were older donor age and prolonged CIT, both well known risk factors for the development of ITBL. Furthermore, XM strength was positively associated with subsequent PCR+ CMV infections. Previous clinical studies have shown acute rejection and active CMV replication to be immunologic risk factor for the development of biliary complications in the context of LT (53-56). Interestingly, a PCR+ CMV infection in immunocompromised HIV positive patients has been known to cause destruction in the biliary tree for a long time, a condition termed AIDS cholangiopathy (57). In a study examining the effects of a CMV infection on rat liver grafts Martelius et al. provided experimental data supporting the role of CMV in the pathogenesis of bile duct injury (58). CMV infection leads to upregulation of MHC antigens and expression of vascular adhesion molecules such as VCAM-1 and ICAM-1 through secretion of pro-inflammatory cytokines (58, 59). Similarly, allograft rejection is thought to induce an inflammatory state at the local level leading to endothelial injury (60, 61). Since viability of the biliary tree depends on the oxygen rich arterial blood supply, an immune-mediated micro-vasculopathy may result in ischemic type injury to the bile ducts, providing a possible pathophysiological explanation for our findings (52, 62, 63).

### Strengths and Limitations

The study compared XM+ with XM− LT using a 1:2 match-pair design. Matching was performed based on the BAR score which has shown to correlate best with post-transplant outcomes compared to other published risk scores (30, 31). Strengths of our study include the prospectively maintained LT database at our center, the match-pair analysis and the relatively long follow-up. Limitations of the present study include the retrospective design and a possible bias concerning the selection of participants beyond the data displayed in the demographics. Despite performing a match-pair analysis in order to guarantee a homogenous comparison group, differences in donor and recipient characteristics did exist between the XM+ and XM− group. The donor BMI was significantly lower, and anhepatic as well as WIT were significantly shorter in the XM+ group compared to the XM− group. This may introduce a bias as a lower donor BMI and shorter ischemia times could imply favorable outcomes. Furthermore, the recipients’ MELD score was found to be higher in the XM+ group. However, the BAR score which, among other factors, includes the MELD score and correlates with relevant outcome parameters following LT was used for match-pair analysis to mitigate potential biases. None of these factors had any significant influence on patient or graft survival in our cohort when performing univariate Cox proportional hazards regression analysis (Supplementary Tables S1, S2) as well as when adjusting for these differences in baseline characteristics in a multivariate Cox regression model (Supplementary Tables S3, S4). Also, University of Wisconsin (UW) solution was more commonly used than Histidine-Tryptophan-Ketoglutarate (HTK) solution as a preservation solution in the XM+ group. UW used to be the gold standard for static cold storage perfusion of liver grafts but preservation with HTK is reported to be clinically equivalent (64, 65). Concerns regarding the higher viscosity of UW leading to an incomplete flush of the peribiliary glands and an increase in ITBL have been voiced. However, these concerns have not materialized (66). Moreover, the type of preservation solution had no significant influence on the development of ITBL in our recipients in univariate binary logistic regression analysis (Supplementary Table S5).

## Conclusion

In the present era of LT, a XM+ has no effects on graft and patient survival as well as postoperative complications. Therefore, our center policy will not change, and we will continue to transplant patients without waiting for XM testing results despite the logistical possibilities offered by the advent of normothermic machine perfusion. A PCR+ CMV infection was more likely to occur in recipients with a strongly positive XM. Together with allograft rejection, donor age and CIT, a PCR+ CMV infection was among the strongest independent predictor for the development of ITBL. Patients with ITBL had higher re-transplantation rates than patients without ITBL.

## Data Availability

Data is available upon reasonable request form the corresponding author.

## Glossary

AIH: autoimmune hepatitis
AS: anastomotic stenosis
AST: aspartate transferase
ATG: anti-thymocyte globulin
BAR score: balance of risk score
BMI: body mass index
CDC: cytotoxic dependent cytotoxicity
CIT: cold ischemia time
CMV: cytomegalovirus
COD: cause of death
CsA: cyclosporine A
CVA: cerebrovascular accident
DCD: donation after cardiocirculatory death
DSA: donor specific antibody
DTT: dithiothreitol
EAD: early allograft dysfunction
ECD: extended criteria donor
ET-DRI: Eurotransplant donor risk index
gGT: gamma-glutamyltransferase
HAT: hepatic artery thrombosis
HTK: histidine-tryptophan-ketoglutarate
IL2: interleukin 2
IS: immunosuppressive
ITBL: ischemic type biliary lesion
INR: international normalized ratio
IQR: interquartile range
LT: liver transplantation
MELD: model of end-stage liver disease
MMF: mycophenolate mofetil
MPA: mycophenolic acid
NAFLD: non-alcoholic fatty liver disease
NAS: non-anastomotic stenosis
PBC: primary biliary cirrhosis
PCR: polymerase chain reaction
PSC: primary sclerosing cholangitis
Re-Tx: re-transplantation
SAB: subarachnoid hemorrhage
SD: standard deviation
Tac: tacrolimus
UW: University of Wisconsin
WIT: warm ischemia time
XM: crossmatch
XM+: positive crossmatch
XM−: negative crossmatch
