# Supplementary material for: Liver Transplantation in Recipients With a Positive Crossmatch: A Retrospective Single-Center Match-Pair Analysis
Source: Transpl Int. 2023 Mar 2;36:11062. doi: 10.3389/ti.2023.11062 (PMC10017503; doi:10.3389/ti.2023.11062)
Supplement: Supplementary file 1 [file DataSheet1.pdf]

Supplementary Digital Content

Table S1. Graft survival – Univariate Cox proportional hazards regression analysis

	HR	95 % CI	P-value
**XM+**	0.941	0.531 – 1.667	0.834
**Donor BMI**	1.015	0.960 – 1.073	0.603
**Recipient BMI**	0.993	0.934 – 1.057	0.836
**WIT**	1.000	0.977 – 1.024	0.973
**Anhepatic time**	0.995	0.973 – 1.018	0.652

BMI, body mass index. CI, confidence interval. HR, hazard ratio. WIT, warm ischemia time.

Table S2. Patient survival – Univariate Cox proportional hazards regression analysis

	HR	95 % CI	P-value
**XM+**	1.330	0.685 – 2.581	0.400
**Donor BMI**	1.010	0.945 – 1.080	0.763
**Recipient BMI**	1.021	0.948 – 1.099	0.589
**WIT**	1.003	0.974 – 1.032	0.849
**Anhepatic time**	0.991	0.965 – 1.019	0.540

BMI, body mass index. CI, confidence interval. HR, hazard ratio. WIT, warm ischemia time.

Table S3. Graft survival – Multivariate adjusted Cox proportional hazards regression analysis

	HR	95 % CI	P-value
**XM+**	0.902	0.501 – 1.840	0.902

**Donor BMI**	1.014	0.954 – 1.077	0.654
**Recipient BMI**	0.975	0.906 – 1.049	0.493
**Indication Tumor**	1.386	0.776 – 2.477	0.270
**WIT**	1.023	0.974 – 1.075	0.367
**Anhepatic time**	0.979	0.933 – 1.026	0.374

BMI, body mass index. CI, confidence interval. HR, hazard ratio. WIT, warm ischemia time.
* Note that the XM+ hazard ratio in the adjusted multivariate analysis is similar to the univariate analysis (0.902 vs. 0.941).

Table S4. Patient survival – Multivariate adjusted Cox proportional hazard regression analysis

	**HR**	**95 % CI**	**P-value**
**XM+**	1.400	0.650 – 3.014	0.390
**Donor BMI**	1.008	0.936 – 1.085	0.832
**Recipient BMI**	1.015	0.939 – 1.106	0.744
**Indication Tumor**	1.345	0.659 – 2.746	0.415
**WIT**	1.046	0.986 – 1.110	0.132
**Anhepatic time**	0.958	0.905 – 1.015	0.147

BMI, body mass index. CI, confidence interval. HR, hazard ratio. WIT, warm ischemia time.
* Note that the XM+ hazard ratio in the adjusted multivariate analysis is similar to the univariate analysis (1.400 vs. 1.330).

Table S5. ITBL – Univariate binary logistic regression analysis

	**OR**	**95 % CI**	**P-value**
**Preservation solution**	0.786	0.207 – 2.987	0.724

CI, confidence interval. ITBL, ischemic type biliary lesion. OR, odds ratio.
